# The particularity of dignity: relational engagement in care at the end of life

**DOI:** 10.1007/s11019-017-9787-9

**Published:** 2017-07-27

**Authors:** Jeannette Pols, Bernike Pasveer, Dick Willems

**Affiliations:** 1 Section of Medical Ethics, Department of General Practice, Academic Medical Centre (AMC), University of Amsterdam, Meibergdreef 15, 1101 AZ, Postbus 22660, 1100 DD Amsterdam, The Netherlands; 2 Department of Anthropology, University of Amsterdam, Nieuwe Achtergracht 166, Amsterdam, The Netherlands; 3 Faculty of Arts & Social Sciences, Maastricht University, P. O. Box 616, 6200 MD Maastricht, The Netherlands

**Keywords:** Dignity, End of life care, Empirical ethics

## Abstract

This paper articulates dignity as relational engagement in concrete care situations. Dignity is often understood as an abstract principle that represents inherent worth of all human beings. In actual care practices, this principle has to be substantiated in order to gain meaning and inform care activities. We describe three exemplary substantiations of the principle of dignity in care: as a state or characteristic of a situation; as a way to differentiate between socio-cultural positions; or as personal meaning. We continue our analysis by presenting cases on dignity in care related to us in focus groups with medical professionals. Our empirical ethical lens is in this paper is to analyse, not the *meaning* of dignity, but the way in which it emerges in practices where it is pursued, within relationships between people, technologies, places, regulations, and the values cherished by or embedded in them. We show that professional caregivers recognize in the dignity of the person they care for their own dignity; giving up on the one implies no less than giving up on the other. This ‘mirrored experience’ of dignity expresses itself in professional’s engagement with the situation. The value of this engagement, we argue, lies not primarily in *realizing* the particular *content* of the values at stake. We point to the importance of engagement *itself*, even if the values engaged with cannot be realized to the full, and even if competing versions of dignity are at stake simultaneously. In this way the caregivers provide us with interesting examples of moral actorship in situations of conflicting values.

## Introduction

The concept of dignity is notoriously difficult to define. There are different approaches to study dignity, but a consensus on its meaning seems improbable. For this reason some scholars have suggested to give up the concept of dignity or the attempt to define it (Pinker 2008; McCrudden 2008; Dieppe et al. 2002). However, for medical professionals who work with dying patients, and to patients and their relatives just as well, dignity appeals to fundamental values of what good care means (Woolhead et al. 2004; Matiti and Trorey 2008). Dignity was also a central concept in developing palliative and hospice care in the 1970s (Lutz 2011). This was a response to the medicalization of death in hospitals, where death was not seen as a dignified ending of life, but as a failure of biomedicine.

The aim of this paper is to further explore the potential of the concept of dignity to support care practices by presenting a pragmatic approach. To do this, we unravel how dignity in care is understood in the literature, to then analyze how it is put to practice in care at the end of life. By analyzing case histories that physicians and nurses presented to us in focus groups, we study how dignity emerges as a concern in practice. We will analyze how this happens through the interactions between the material setting, the activities of the participants, and the values embedded in or cherished by them. We will show how in these concrete situations it is not the determination of the *meaning* of dignity that leads to problems. This meaning is often clear to those present. What poses problems is rather the crafting of dignity in a situation that cannot be influenced, or when opposing values clash. How does dignity at the end of life emerge in the relationships between people and situations, values and givens? What material and social elements contribute to this result? What does this teach us about the moral potential of dignity as a concept to improve care?

## The approach

We take an *empirical ethics* approach. Empirical ethics refers to the (ethnographic) study of normativity as it emerges *within* and *from* practices, rather than being added to them from outside (Pols 2015, 2017; Willems and Pols 2010; Mol 2010; Willems 2010; Pasveer and Akrich 2001; Sharon 2016). Normativity refers to the different ‘forms of the good’ that appear in practice (Thévenot 2001). Goodness is a ‘loose’ or sensitizing concept and serves as a pointer to observe the situations in which we are interested (Pols 2015). It gets its meaning and shape through empirical study. Goodness can take many different social and material shapes. There may be norms to follow, values to put into practice, machines to switch on or off, ideals to strive for, tastes to appreciate, medication to prescribe or withdraw, or prescriptions to command. We hence study forms of the good as well as forms of doing good as they are part of our socio material world as well as of our thinking.

Ideals such as dying-with-dignity take their shape in care practices amidst different notions of what is good to do. All participants, social and material, play their part. There are doctors and nurses, patients and families, but also the patient’s body responding to treatment, as well as the medical and everyday technologies that influence the dying process. The specific locations where people die, such as the hospital or the home, also influence the care that unfolds (Hockey et al. 2010). The patient may hope to be cured after all, the morphine pump reduces pain, and the nurse may be anxious to bring in the family if death is near, all in the same situation. By analyzing the alignments or tensions in the interactions between these participants and the goods they pursue or embed, we aim to show how situations unfold in which dignity is a concern.

## On dignity

Before discussing the focus groups, we briefly map scholarly approaches of dignity in the literature. Philosophers, ethicists and scholars of law often analyze dignity as a principle. This principle is abstract and universal, but inherent to human beings.^1^ In actual care practices, however, this principle has to be substantiated in order to gain meaning and inform activities.^2^ Dignity comes to refer to particular values in particular situations. We continue by describing three popular and scholarly substantiations of the principle of dignity: it is regarded as a state or characteristic of a situation (‘The way he died was (not) dignified’); as a way to differentiate between socio-cultural positions (‘To be autonomous is more dignified than to be dependent’), or as a category that is substantiated through individual meaning making, for example in qualitative social scientific interview research (‘For me dignity means…’).

### Dignity as a principle

For philosophers and lawyers dignity is a foundational principle (e.g. Kateb 2011; Barilan 2012; Waldron 2012; McCrudden 2008). Western, particularly Kantian philosophers and scholars on human rights find themselves on solid ground when they interpret dignity as the founding principle for the universal rights of man. The principle is that all human beings *have* dignity, simply because they are human. Protecting this *intrinsic* (Leget 2013) or *inherent dignity* (Nordenfelt 2004) would mean to protect humans and humanity itself. Every human being, everywhere on the globe has dignity. It is a universal ethical principle. This includes oneself and others, and, in Kantian philosophy, briefly, means that human beings should be treated as ends in themselves, where the worth of a person has no price, and is an inalienable property.^3^

In this way dignity is taken to be an intrinsic, given part of being human, whatever characterizes the specific individual. One could say that it is a concept that is empty of particular content, it is unconditional. This resonates with Rosen’s (2012) analysis that dignity ‘has no coherent meaning of its own’, but may be seen as a receptacle of different ideas.^4^ Treating a person as an end requires treating that person as having a dignity or worth that is beyond the worth of what a particular person does or is. Phillipa Byers argues that dignity is independent of one’s own goals and purposes, yet it gives value to these purposes (Byers 2016, p. 63). She argues that dignity is the ground of any other kind of value (ibid., p. 63) and is the sole reason for duty or moral activity towards others. There is no purpose to morality other than that of paying respect for the other’s dignity.^5^

When dignity is seen as inherent worth, some scholars state that dignity is something that can’t be made, but that can be violated: ‘… health care professionals cannot confer on patients either dignity or death with dignity. They can, however, attempt to ensure that the patient dies without indignity. Indignities are affronts to human dignity …’ (Allmark 2002, p. 255). In this way, there may be situations that do not honor intrinsic human worth. However, to describe situations where this happens, the universal principle of dignity needs to be made concrete. And this is one problem with understanding dignity as an abstract principle; as such it is difficult to use to improve care practices, other than through an unspecified appeal to care that is worthy or humane. We all can truthfully declare that we cherish the dignity of a person and subscribe to the importance of treating people with respect. Yet it is unclear what the consequence of this would be in practice.

The unconditional and non-descriptive character of dignity as a principle is both its force and its weakness; it appeals to many and nobody could be against it, but it refers to different values in particular situations. Tensions in practice emerge when there are differences in how to understand a respectful approach, or how to best interpret dignity. It is these empirical substantiations that we will now explore by describing three common ways to use the concept of dignity.

### Dignity as a state or situation

One way of understanding dignity in a concrete way resonates with the idea of dignity as a state that can be violated. Dignity then becomes a characteristic of a situation or state a person is in. This happens for instance when there are protests against particular situations that are described as undignified. One example from the Dutch context is that an elderly lady living in a nursing home was observed while ‘urine was running along her ankles’ (AD 4-11-2014). This was attributed to bad care. The incident followed an earlier public outrage about ‘pajama days’, where the residents of nursing homes were not washed and dressed on particular days due to a lack of staff (Trouw 29-6-2006). The situation was described as one that violates dignity, and hence was unacceptable.

Julia Lawton’s paper (1998) describes a situation that could ultimately be defined as undignified in order to show how society deals with the limits of dignity. She observed people in a hospice, where they were dying from cancer. In plastic descriptions Lawton theorizes a loss of dignity as a loss of boundaries, for instance in situations in which bodies were leaking bodily fluids. These unbounded bodies, literally, had holes in them through which fluids came out and merged in undesirable ways. The hospice, Lawton argues, served as an institution to exclude these disintegrating bodies from society. In these examples dignity or the lack of it is characterized as a state, and is often used to expose bad care situations. It may also describe desired and dignified situations. ‘It was in the style in which she had lived her life.’ ‘It was a death without suffering and pain.’ ‘It was a peaceful death.’ The idea is that it is possible to come up with definitions of situations that are dignified -or not.

### Dignity as a way to describe social differences

Another way in which dignity is substantiated is when it is used as a way to describe social differences. More, or specific dignity is granted to some persons. For philosophers, the example to stumble over was already given by Cicero. Cicero asserted that one citizen can be regarded as having more dignity than another, because of their social position, status and responsibilities. Meritocratic principles or matters of status categorize some citizens as better than others. In Boltanski and Thévenot’s (2006) formulation, some citizens contribute more or more important things to the common good than others, granting them more dignity.

Appiah (2010) develops the possibility of differences in what he calls ‘honor’. He distinguishes respect for ‘peer honor’, which is recognized when a person shares a particular status with others from the same ‘honor group’, from respect for ‘competitive honor’, which one gains by excelling in relation to a specific honor code. In this way, differences between the dignity of people and social groups are articulated (see also Waldron (2008, 2012) on ‘rank’).

This notion of differentiated dignity clashes with the idea of an inherent universal principle. Ultimately, it would pave the way for a distinction between citizens and, say, slaves, by deserting the ideal of equality. Still, debates about citizenship, in particular in relation to refugees and migrants, show that principles of dignity can be and are interpreted in a different way for different people (see also Foucault and Faubion 2000; Pols 2013b). Different rights and living conditions are ascribed to different groups of people, allowing some and not others to work, go to school or participate in other social institutions.

Another way to look at dignity as relating to social differences is through the concept of social dignity (Leget 2013; Tauber 2014) or dignitas (Pols 2013a, b), or again: honor (Barilan 2012; Appiah 2010). Dignitas and honor are linked to concrete aesthetic values, as a way to connect the discussion about dignity to notions of ‘the good life’. Aesthetic appreciations of the good life take the daily life and the relationships of people as objects to which, in Foucault’s words, characteristics can be attributed that are otherwise reserved for Art: life (Gr: *bios*) as a beautiful work (see also Foucault and Faubion 2000, pp. 61–62).^6^ Hence we may speak about a good life or a good death in aesthetic terms: a colorful or courageous life, a beautiful ending.

This aesthetic view on dignity points to the different ways in which what people value and appreciate links and separates individuals. One person may have a preference for the same food as another, but may not be interested in the same person’s passion for rock music. Different aesthetic genres organize different people in different ways, by attracting or repulsing people, be this by habit, passion or education (Meyer 2009; Meyer and Verrips 2008).^7^ This perspective is also interesting when looking at dignity in end of life situations. Burial rituals for instance, are favorite objects for anthropologists to study: they are different everywhere. These differences relate to cultural traditions for aesthetic genres, including one of individualization. What dignity is, is substantiated through social influences.

### Dignity as personal meaning

Even more specified and particular is the understanding of dignity as an articulation of individual values. This is the dignity that emerges in social scientific studies that ask people, often in situations of great need for care, what they find dignified (e.g. Chochinov et al. 2008; Clement 2002; Kendall et al. 2007; Masson 2002; Pattison et al. 2013; van Gennip et al. 2013; Oosterveld-Vlug et al. 2014). This literature does not aim to deliver the ‘final meaning’ of dignity. Yet it shows how people understand dignity in their own situation. The informants may refer to principles, social values, states in which they are and ways they are being treated. But ultimately, dignity is substantiated by individuals in particular meanings. An example from our focus group discussions shows this nicely—if the reader will excuse us for running ahead of the empirical analysis.^8^

Hospice nurse: You don’t just look at medication, but regard all aspects of a person. You always want to do that, but particularly when someone is dying. You have to find out who this patient is and what is the most important concern to him or her. This is what we [caregivers] do wrong so very often: we make that up for the patient. If someone is in bed and moans every now and then, we think: pain! And rush off for pain medication. But in the palliative situation you have to think: what is most important for you now? And you’ll be surprised. Because one person will say: ‘If only there is a solution for my cat after I’m gone’. Another is afraid of pain, yet another wants to go home, some people want you to arrange some practical things, and so on, and so forth. You have to *ask* them. You don’t need to fly in a palliative team for that. Somebody just has to ask.

This approach is crucial when it comes to providing good care for individuals. But this specificity is also a weakness. It may make it more difficult to understand the enigmatic notion of dignity on a conceptual level. Because it individualizes dignity as a set of personal values, it runs the risk of losing sight on the social dynamics that inform notions of dignity. When, for instance, autonomy and independence are highly valued in a society, it will be viewed as problematic to live in a situation in which one is dependend on care. Matters of how to live and how to die are not only individual questions, but are social questions as well. Values may be expressed by individuals, but simultaneously point to social and political questions. Controversies about the acceptability of euthanasia are a point in case, as they differ from country to country. Conceptually, dignity cannot be reduced to its substantiations as individual preferences, even if such an understanding is crucial in actual patient care.

## The focus groups

So we have dignity as a general principle without concete directives, but that is valid for everyone everywhere. It needs protection because it can be violated, as a state or situation somebody is in. There is dignity as a way to refer to social differences and different understandings of honor between people. And there is dignity as perceived by individuals. The concept moves through many registers between the most abstract universal and the most concrete particularity. We will now turn to the focus groups to analyse how dignity emerges in care practices for people who are dying. Our attempt is not to give another *interpretation* or substantiation of dignity, but to see how the various forms it takes come into being, where possible tensions emerge, and how the actors respond to these. In this way we attempt to add an extra venue for exploring dignity in care.

We report on the results of two focus groups, one with five medical doctors, the other with six specialized nurses. Three of the doctors and five of the nurses worked in an academic hospital in the Netherlands. The doctors’ group (4 women, 1 man) consisted of a neurologist, an oncologist, a pulmonologist, a general practitioner and geriatrician. The latter two practiced outside the hospital. The group of nurses (5 women, 1 man) consisted of three pulmonology /gastroenterology nurses, a hospice nurse (from outside the hospital), a neurology nurse, and a neurology nurse from the hospital’s palliative care team. The groups were organized and chaired by the authors. The sessions lasted 2, 5 h each, were recorded and transcribed.

We asked the participants in advance to think about situations concerning end-of-life care they had participated in and in which dignity had been at stake. In this way we asked them for detailed observations of clinical situations concerning the end of life, and to adopt an ethnographic gaze on their own practices: to describe their activities, feelings and thoughts of the participants, as well as the material context. This is not a ‘proper’ ethnography in the sense that an independent observer maps positions and events. In the cases we cite, the points of view from the dying persons and their families are not always as extensively reported because we did not speak to them. This method did, however, provide us with rich cases that show where *professionals* felt dignity was at stake and how they acted.

## The focusgroups in practice

It was clear in both meetings that the question to talk about dignity evoked concerns that were deeply felt by the participants. The clinicians took dignity, particularly in end of life situations, very seriously, even if it was sometimes hard to find the words to talk about it. The conversations were intimate, respectful and intense, with the professionals presenting cases that were close to their heart. Day to day clinical language did not fit well to describe issues of a good end of life. Much in line with the results of our other studies, dignity was often described in *aesthetic* terms, as ‘beautiful’ ways to end life, as a ‘proper’ way to go, or as ‘horrific’ or ‘sad’. At the same time the intensity of the conversations showed the moral significance of values related to dignity for dying people.

The professionals in the focus groups supported the analysis by their active engagement with each others’ stories, by asking questions, highlighting points they thought relevant for dignity, and occasionally providing comfort to a colleague re-living an intense situation. As good clinicians they were not moralizing in the sense of *judging* one another. Cases about dignity were cases that *mattered* to them and to being a good professional. It is this commitment that provided pointers for our analysis, as we will now develop.

## A place for dying: death in an intensive care unit

The desire to die at home is shared by about 75% of the (Dutch) population (Akker et al. 2005), but Koekoek (2014) found that 22% of the people wanting to die at home, actually died elsewhere. The place that is generally believed to be highly unfit for a dignified death, is the Intensive Care Unit. The first story shows how dignity emerged in care for a patient who was dying in a hospital.

A man in his early fifties was on a palliative trajectory with incurable cancer. He had sold his company, and his oncologist described him as sad but accepting his fate. A last possible treatment option came up, which emerged after the treatment team discovered they had misdiagnosed the type of cancer he suffered from. The team and the family discussed all options, and this patient was admitted to the hospital because he wanted to give this treatment a try.

Oncologist: His condition was really bad, he depended on oxygen … well, he really was almost dead. But he still had an almost 50% chance of being cured, you know, so you think it is worth trying. But we also told him that he might end up in the Intensive Care (IC) because his lungs might fill up as a result of the chemo. And this is what actually happened. So in the course of that weekend he was brought to the IC, put on a ventilator, we tried to continue the chemo, but then he developed kidney-failure, he needed cardiac support, it all went bad. So in the end we decided, all of us, the IC physicians, the family, his daughters, to stop the treatment. So when we are talking about dignity… I don’t think an IC is a dignified place to die in. You really want to spare people that. But his death was very dignified nevertheless. And I’ve been thinking: why? It was his family that stood around him, literally. He was in a separate IC cubicle, and I’ve never seen it so filled with people. All his loved ones where there to support him. And then the moment came that we shut down the monitor, because we tended to look at the machine all the time to see whether he had died … that was beautiful.

Other doctor: Very good that you thought about switching off the monitor.

Oncologist: I admire the family for being able to look through that, to ignore the devices. And what I found beautiful too—funny, I use the word ‘beautiful’ all the time!—was that when this man died there was no white [professional clothing] to be seen. It was him and his family and friends. […] It was a dignified death.

The oncologist stresses the absence of obtrusive medical aids and white coats. The switching off of the monitor moved the attention of those present from a situation of a heart that would eventually stop (signifying the failure of curative medicine) to the situation in which everyone who cared for the patient was around the bed. When his body was giving up, the life-supporting situation turned into an apparatus that was inconsistent with a dignified death. Machines that administer medication and monitor life were switched off. The normativity of the place mattered to dignity. The hospital comes with an infrastructure that is oriented towards cure and life, not palliation and death. This place had to be re-ordered by foregrounding the family and retreating devices and white coats. *Technologies* played a crucial role in both the dis- and re-appearance of dignity. There was the chemotherapy, the life- supporting technologies in the ICU, the trajectory towards cure. Then there was a practical and moral reshuffling when it was clear the end was near.^9^ The setting transformed from one of survival into one of peaceful dying. *People* were important too: the presence of the family, the withdrawing professionals, and the patient himself, who gave the treatment a try.

The story shows how dying with dignity is not given with a certain setting, notwithstanding how strongly the situation is pre-programmed to achieve cure or survival. The people, machines, the history of treatment all interacted to stage dignity in the last phases of this man’s life. All these elements were newly aligned in this situation, making the doctor assess it as a dignified death, notwithstanding the things that had gone wrong, and the place that made dying with dignity difficult. Dignity emerged as a relational achievement.

## The mirrored experience of dignity

Our second, more troubled story was told by the general practitioner and concerns a man in his sixties who was terminally ill with lung cancer. Here, the different elements could not be aligned in a way that was satisfactory for the participants.

GP: He [the dying person] wanted to die at home, and he wanted to be cared for by his partner and her daughters. And his partner also wanted to do that for him. Complicating was that he had quite some debts, and the woman was illegal, she did not even exist. It was winter, and his situation was getting worse. He received pain medication and home care for he was in bed most of the time. At that point their gas and electricity were shut down because of unpaid bills. So the electric high-low bed did not function anymore and because of that the home-care nurses were not allowed to go there anymore. You know, labor laws and all. Notwithstanding the electricity we borrowed from the upstairs neighbours to move the bed, home care workers were not allowed in anymore. The house was lit with candles and heated with a gas-stove. My assistant occasionally helped out to clean the bed. And this man refused to be taken elsewhere because he knew: then my partner has to leave the country. This would eventually happen anyway, but we did not know how to manage this. We tried to convince the municipality to put the electricity back on, but they could or would not do anything on such a short term. And so this man spent the last weeks of his life in a very marginal situation: in a cold house, candles that seemed nice but were not, with insufficient care, and insufficient support for his partner who was very sad and desperate about her husband dying and her own future being so insecure. We went there a lot to do whatever we could. But it all felt very wrong. The only good thing was that they were together, but in a setting we felt was very undignified to die in.

It is clear from the description that all participants were very concerned with the (digntity of the) situation. There were important values at stake. The situation shows the impact of the socio-economic situation of the dying person (the debts) and the partner (having no legal status to be in the country, even in the face of her partner dying), the material situation of the cold and unlit house and the abandonment by the official institutions (home care, the housing company). There was support from the GP practice and the upstairs neighbours.

The attempts to align the relations between different elements are visible again. *Place* crucially mattered. The house provided a good place to die and safety for the partner. But without gas and electricity the place became a bad place as well. *Technologies* mattered, the everyday technology of electricity, gas stoves and candles, as well as the medical technology of the painkilling and the high-low bed. Rather than their mere presence or absence, the specific roles these technologies played was important. The withdrawal of electricity rendered the house dark and cold. Legislations regulating working conditions and conditions for citizenship made a strong mark on the situation.

*People* played an important part in the achievement of at least some respite: the patient, the partner, the daughters, and the GP and his staff. The partner had a double role in the emerging situation. On the one hand the patient could not be transferred to a hospice or nursing home because that would have jeopardized the possibility of her presence. On the other hand their relationship and their care for each other were, in the GP’s story, ‘the only good thing’. Although ‘home’ is where most people prefer to die, it can only provide for a good death when it is firmly supported by relations with specific other places (health-care institutions, electricity companies), technologies (bed, medication, electricity), regulations and people.

Did dignity fail to emerge, as the GP suggests, where the lack of dignity is a characteristic of the situation? From our analytical outside position and relational perspective we see something else emerge as well. Notwithstanding the difficult situation, the GP and his staff did not abandon the patient and his partner. They assured whatever care they were capable of providing, ranging from calling the municipality, visiting often, and taking care of clean bed linen. There was pain, but also pain killing. The value experienced as most central was achieved: the patient died in the presence of his partner, even if the circumstances were terrible. From our analyst point of view we could see the *engagement* of the people in this situation as remarkable in relation to dignity. This engagement to pursue dignity, even if it was clear that it could not be fully realized, shows something crucial about dignity. The caregivers’ relational engagement was not so much about *realizing* the particular *content* of the values at stake—they could not achieve this. But is an action good only if the good is achieved? We suggest that it is the engagement with these values itself that signals dignity, even if these values cannot be realized to the full. We can recognize dignity if the good is of such importance that it is pursued and not given up on, even if it cannot be attained, or not completely.

By not giving up on the patient and his partner, the caregivers showed how their own dignity was mirrored in the dignity of those they cared for. To give up would also have meant a loss of dignity of *themselves*. Their worth as professionals depended on the engagement with the situation of the patient and his family. If they would give up caring for the situation, they would give up on their own dignity. An example from the hospital brings this mirrored experience of dignity to the fore even more clearly.

Hospital nurse: When the famliy arrived early that morning, Mrs. Moritz was already quite far away. She slept, she was attached to a morphine pump the day before. She was at ease and her family thought so too. But at 6.30 her husband comes to get me and when I go over I see she is choking. It looked like an insult, she was very restless and looked troubled. So I called for a doctor who came right away. And when I got back to her, her family had started demanding things. My colleague was apparently not aware that she was what we call a ‘code D’ patient—which means she would only receive palliative care and no treatment. My colleague had fetched a couple of devices to regulate blood pressure and all that. So I said to her: ‘We’re not supposed to do that anymore’. And the doctor had started with [sedative] to put Mrs Moritz at ease. She had stopped choking. But her family said that the mucus that she brought up bothered her and they wanted me to do something about that. I told them that putting in a drain would only deteriorate the process, and that I could only clean her mouth a bit. And then her son got really aggressive: ‘Just do it! Go get it! Go!’ So I am running around as if I was dealing with an acute case and I go fetch the drain and I’m putting the device together and I say to myself: ‘When all this quiets down I need to discuss this with the family’. I did not feel good. And all of a sudden they say: ‘It looks as if she has stopped breathing’. So I go and see and she had died. I said: ‘I’m calling the doctor’. They shouted: ‘Go then! Don’t stand there like that!’ So I go fetch the doctor and we walk back and the son comes out of the room and he says: ‘She died and you should have done something!’ It was terrible… [cries]… For in the end this woman died all alone, there was nobody looking after her when she died.

The pain of the narrator of this story is that she hadn’t been able to pursue her engagement with the dignity of the patient. Instead, she was pushed around by the angry and anxious family, rather than staying true to what she thought was of value. She would not get a chance to make it up to the patient—there is only one chance to die well. The power of this story is that it shows how the dignity of those cared for is mirrored in the dignity of those caring, and how it moves the caregiver. If the caregiver would ignore the threatened dignity of the patient, she would, in her own eyes, loose her own dignity. She would not be a worthy caregiver if she did. She has to engage with the situation of the patient, or she would give up on deeply felt values of worth. Ultimately, both her and her patients’ dignity (or worth) are at stake. And ultimately, the engagement of this caregiver shows her emotions as a concern with dignity that she will not give up on, even if she could not realize it this time.

## Confliciting dignities

So there is the engagement of caregivers who recognize their own dignity, indeed in the sense of a general feeling of worth that is specified in a particular situation, in the people and situations they care for. The last story analyses more in depth how professionals dealt with conflicting understandings of a dignified death. The story is from the geriatrician.

Geriatrician: it was a man from Northern Africa, 70 years of age, who was admitted to the somatic ward of our nursing home because of an increased need of care. He had very advanced Parkinson’s, severe swallowing problems, a feeding tube, and he had suffered a number of pneumonias. He was completely immobile, bedridden, incontinent for urine and faeces. And he was hypertonic [having involuntarily tensed muscles], so he lay there like a person with progressive dementia, with his knees pulled up towards his face. We estimated he would weigh about 35 kilos. We saw a patient who should have been admitted long before, but who had been taken care of at home with a little bit of homecare. He could not speak anymore, so his cognition and mood could not be judged. At first he could still say ‘thank you’, but his motor skills failed and maybe he had little Dutch language left. In the end he expressed himself only by moaning, and that really chilled us to the bone. You could hear it from the end of the corridor. On average once a week he pulled out his nasal feeding tube. It was unclear if he did this on purpose, or if his nose was itching, or if he had had enough of it and wanted to stop. When we put the tube back we had to constrain him with a couple of people. Well, the nurses still vividly re-live these situations. He appeared to be choking, he turned blue, it was terrible. As far as we were concerned, we would not apply the tube again. But of course this was a decision moment [This refers to a legally stipulated moment where patients are allowed to make their own decisions.]. We thought it an undignified, terrible treatment that may have prolonged his life, but certainly also his suffering.

And we discussed how to deal with this. You try to reconstruct a will, that is one of your ethical principles, and we tried to find out if this was indeed the reconstructed will of the patient, as his son claimed. The last time the son had talked to his father, about six months ago, father had told him that he wanted treatment if this would keep him alive longer, even if the treatments were not so nice. We couldn’t check this with the father anymore, and we did think that the son had taken it too literally. But the son found that this should be the guidance for the treatment, even if he saw that his dad was very ill and very handicapped. The son felt he acted in accordance with his father’s will by asking us to continue with the tube feeding. The son respected our worries and resistance, but he insisted that reinserting the tube was in accordance with his father’s will.

What to do? The son was clear that medical treatment and support should be prolonged, as this was the wish of his father.^10^ He insisted, and this was also his right, as family members represent the patient when they are not able to speak for themselves. To the son, this was a dignified ending of his father’s life: staying true to his wish. We lack empirical detail on the position of the son, but is seems he shows a different mechanism for his engagement with dignity. He is not observing and empathizing with the elements that make up a concrete situation in which the patient is in distress or otherwise threatened in his dignity. He is rather applying the norm that one has to honor the wish of the dying person—even if this leads to concrete unpleasantness. The wish is taken to be more important than its consequences.

Clearly, the team had very different feelings that were no less deeply felt. The dignity of their patient was at stake, particularly when they had to hurt him to replace the nasal tube. For both the son and the professionals the substantiation of dignity was different (respecting a wish versus making someone comfortable), but also the mechanism to substantiate it (empathy or applying a principle). Yet both shared the engagement to pursue the values they deemed essential to the dignity of the dying man, and - through the mirroring function of dignity—to their own.

What to do when two deeply felt understandings of dignified treatment are so clearly opposed? Rather than a delineated moment of decision making, the situation unfolded practically in different ways. The son started taking his father to the emergency ward on the other side of the street for a replacement of the tube. The emergency doctor re-inserted the tube without much trouble. He was a strong man, and when the geriatrician asked him about it, he did not report any ethical concerns. He simply did what the son asked him to do: a routine medical act.

Then the family agreed to the increase of pain medication and sedatives, so the moaning stopped.

Geriatrician: What helped was that the nursing team kept looking for options to make him more comfortable and to preserve his dignity. They paid a lot of attention to washing and dressing him, preferably with two nurses so as to do it really sweetly and without hurting him. They talked to him, covered him so as not to let him lie naked, which is maybe even more important for this man than for others. We also asked the family to bring African music so as to make him feel at home as much as possible. The nurses also took a lot of time for each other, to create room to discuss their frustrations. They were allowed to state a limit when they did not want to insert the feeding tube any more. By taking care of each other they could also care for him better. It also helped that twice a week his imam came in to pray with him. That felt good for us, it showed care and connectedness.

In this example the participants accommodated what seemed to be irreconcilable differences on the issue of dignity, an issue that was crucial to each. By practical solutions and by investing in what everybody though was really of value, they shaped a situation that was livable for everyone, even if it was not perfect. The tube was placed by an expert without bad feelings about placing it. The son contributed by taking his father to the emergency care, relieving the team of what they felt was maltreatment. Culture and religion played a part, both in the weight attached to the fathers will, and in the ways to relief his suffering through music and prayers.^11^ The nurses invested in what *they* found important in care in two ways. First, they added good experiences for their patient to bad ones: careful washing, music, prayers. Second, the nurses invested in one another. This is important when giving care that is not dignified also jeopardizes the dignity of those administering it. By caring for one another, the professionals made it possible to work in circumstances where dignity was challenged—their patient’s dignity, and hence also their own. The mirrored experience of dignity was acknowledged and cared for, even if value conflicts could not be solved.

## Co-laboring dignity

In this last situation tensions emerged not primarily between conditions and ideals, but between explicit understandings of what was needed to honor dignity. No compromise was possible, due to the mirroring of one’s own dignity in the dignity of the other. The values dignity stood for could not be compromised or given up on. They matter to the caregivers, and are crucial to their enagement. The anthropologist Marisol de la Cadena (2015) catches this idea of working with different understandings and valuing in the notion of co-laboring. She works with a Quechua speaking Peruvian informant, to learn about the social struggle against rich colonizers. Her informant has very different world views than she has, as an academic trained in North America. Her informant, for instance, speaks about mountains and lakes as ‘Earth Beings’, critters endowed with moods and a will. She does not want to reconcile different ideas about mountains by translating one to the other. Marisol de la Cadena does not want to accept that mountains and lakes are earth beings, but also does not want to tell her informant this is merely his *belief* while her world view stands for how things *really* are. Instead she wants to keep both worlds intact, and not reduce the one to the other. As with the conflicting notions of dignity, she attempts to co-labor, to work together while accepting different understandings of the world. And, as both her book and our last case study shows, this demands practical work-arounds and mutual acceptance of incommensurability. Translated to our topic, this would mean a co-laboring to craft a situation of dignit*ies*, plural. In our last case, for instance, the dignities were ‘to respect a person’s last wish’ versus ‘to treat a person well and inflict no pain’.

It is this engagement to co-laboring, this preparedness not to give up when the good cannot be achieved as one would have hoped for, that is crucial for dignified care in settings that brought coldness and candle light, or in conflicts about what is the most dignified approach. This engagement is not merely about deliberations: some bridges cannot be built, and material actors certainly do not deliberate. There is no consensus, choice or compromise.^12^ It is an attempt to stay in relation, by keeping the values felt as important active within these relationships, even if they would never be attained. This engagement emerges as crucial for dignified care. Engagement with dignity, then, is the opposite of indifference. It is not achieved by enforcing rules—this would make a situation of co-laboring difficult or impossible as one ‘solution’ is chosen over another, while risking the elimination of the affective and introspective process of mirroring. This might invite for indifference or for the risk of bureaucratization of ethical issues (Pols 2003). Only through these motivational processes and use of their moral compass can caregivers be expected to provide dignified care. Conditions for dignified care, then, need to accommodate and stimulate processes of mirroring to give space to the engagement of caregivers to pursue what they value as dignified care.

## Conclusions: the particularity of dignity and the improvement of care

We analysed dignity as it emerged as a concern in relations between people, technologies, places, regulations, and the values cherished by or embedded in them. Through this analysis we learned about the relational character of dignity and its ‘mirrored experience’, as the dignity of the caring person was interdependent and evolving with the dignity of the person cared for. Crucial was the feeling that the particular values dignity related to were essential to people, leading to a necessity to engage with these values. Values relating to the concretisation of dignity are values that cannot be ignored without compromising the one who values. They have to be engaged with at the cost of jeopardizing one’s own dignity.

From our analytical (outside) perspective dignity was not the universal, inherent, yet ‘empty’ principle, but neither did the concept dissolve in particularities. We found an empirical generality^13^ in peoples’ engagement with dignity and the values it refers to. Engagement, we argue, is the key term to understand dignity. It is made concrete in different ways, but it becomes apparent when people engage with it. Through these engagements dignity gains its characteristic as a general particular. We all engage with values we hold as crucial, but what these values are may differ between us. Crucially, people did not emerge as *having* dignity, but as beings that appreciate and value, and engage with these values in concrete situations. The ‘good caregiver’ that emerged from our analysis was fallible, but saw his or her own values reflected in the relationship to their patients, families and material situations.

One could see this linking of one’s own situation to the situation of others as something that ‘comes naturally’ to caregivers, but one can also interpret the assessment of dignity as aesthetic and moral ‘technologies of the self’, in the foucauldian sense, as a ‘technique’ for improving the self in relation to others. As a technique it is not an ethical prescription, but an aid to support, orient and substantiate one’s engagement with goodness. We analysed two techniques of the self: one that worked through empathizing with people and situations,^14^ the other through seeing that the last wish of a person is honored. Because the values at stake were related to dignity, the care for others simultaneously signified care for the self.

What about the dignity as understood by the dying person? In our cases most of the dying persons were no longer active, but they had been engaging with valuing their own situation. They went for that last treatment option, wanted to die with their partner close, left things to their family or formulated a final wish. But again, they did this in relation to others, such as their families, doctors or legal situation. Dignity in practice emerges as a relational concept; it is mirrored by others, hence it exist in relation to others. Dignity, then, has little meaning as a descriptive attribute of an individual alone, mirroring itself (‘I have dignity’, regardless of what happens around me). The concept signified an ethical relationship between selves and others. It is not reducible to the judgments of one person. Dignity emerged in a social context in concrete relationships. It was not a situation or state, but a process of creating and maintaining ethical and aesthetic relations through engagement and mirroring.^15^

What are the ethical implications of this analysis for improving care? As moral actors the caregivers in our study showed what was precious to them. They did not do this by *realizing* the values related to dignity, even if they did stay true to what they valued. They kept pursuing these values in situations that were difficult or that threatened to compromise these values. They co-labored with different approaches to dignity, leaving the values of others intact, not dismissing them or reducing them to their own.

In this way they did not act as fundamentalists who attempt to push values that are crucial to some, and indifferent to others, on those others. Also, they did not merely follow rules that prioritized some values over others, but co-labored with rules if they felt dignity was at stake. Co-laboring did not turn them into relativists either. As practicing moral actors they stayed engaged with the situation at hand, as it emerged in its concreteness. These differences asked for different responses and activities were developed, on the spot, together with those present. If needed, the caregivers went out of their usual routines in order to try to make things better. Hence there was no ‘debate’ that was settled or ‘decision made’, but there was a practice where different goods were pursued simultaneously or co-existed in tension with bads or with alternative goods.

The caregivers in the study hold up a moral mirror for us on how to deal with value differences. They point us to ways in which care practices can indeed be supportive to dignity in emerging situations, by caring for patients through caring for the caregivers. This can be done by supporting and facilitating the caregivers’ capacity for mirroring their dignity in the situation of those they care for, and by supporting their engagement with the situations they are in. This will not guarantee perfect outcomes, nor is engagement in itself sufficient; professional skills and supportive conditions are also needed and values may differ. However, building supportive infrastructures for professional engagement may guarantee a general commitment to good care in situations where concrete values are in tension.

## Footnotes

1 There are interesting discussion on including *animals* as having dignity (Singer 1995), and then why it would have to stop with animals (Abrahamsson et al. 2015). Since the global problems we are facing appeal to interconnectedness of people and other life forms, then to individual agency, this discussion is timely. In his discussion on the relationship between dignity and rank Waldron (2008) argues that dignity does not simply generates an egalitarian approach, but ‘levels up’ the rank of all human beings into a ‘society with just one cast’ or class (p 71), creating a stratification to separate humans from other beings. We have to leave this debate aside in this paper.

2 see Appiah (2010), Rosen (2012) and Waldron (2008, 2012, 2013) on this argument, and Pols 2013a, b.

3 See Rosen for an excellent interpretation of Kant’s complex position.

4 This is not Rosen’s own position. He uncovers different historical strands of meaning of the term. His main concern is, however, comparable to ours: even if we have grasp of the principle, it is unclear what ways of acting could or should be inferred from it.

5 In Byers (2016) paper it is ambiguous if the capacity to set goals and being autonomous is not a condition for being included in the species that have dignity. We have dignity because we set (rational) goals, but our goals gain worth because we are dignified.

6 It is important to see that in Foucault’s work with ancient philosophy the ethical, aesthetic and the true are not separated, but are all present in the same situation. There are not separate domains for either. Foucaults major interest is in the relation between the speaking of the truth in relation to the good life, leaving open many questions about the relationship between ethics and aesthetics here (author, in preparation).

7 Or the big sociological categories of class, gender, and so on. See e.g. Bourdieu 1984.

8 But see also how the general principles of respect and dignity are immediately individualized in the NHS formulation: “Every individual who comes into contact with the NHS and organisations providing health services should always be treated with respect and dignity, regardless of whether they are a patient, carer or member of staff”.

9 See Pasveer and Akrich (2001) for a similar argument concerning ‘natural’ childbirth.

10 Kaufman (2005) writes about the asymmetry of considering the removal of life-supporting technologies as an act of actively choosing the patient’s death or taking God-like decisions versus prolonging treamtment that needs no such interventions. Stopping treatment is an active way of ending life, whereas prolongation of treatment seems to be ethically right, demanding no further justification.

11 See Candib (2002) for an analysis of how the values of ‘autonomy’ and ‘truth telling’, central values in western medical ethics, may clash with values in other cultures and ways of treating people; Blank (2011) for an analysis of the relative lack of knowledge of these different cultural frameworks. Our example shows that cultural differences are not clearcut.

12 The Dutch technique of ‘polderen’, discussing about conflicting standpoints until a compromise is reached here appears in a less benign light.

13 It is not universality, as this is not a theoretical generalization, but one that stems from empirically analyzing particular situations. It is present in the situations analysed. It is not a prescriptive generality, but an empirical one; it can be falsified by finding the popperian black swan or an ethnographically ‘different practice’, a situation where caregivers do not engage with matters of dignity, do not value or appreciate, or in situations where less crucial values are at stake than in situations where people die.

14 This bears resemblance to Adam Smith’s importing of the ‘impartial spectator’ to understand the moral meaning of a situation through sympathy (empathy).

15 This is not to say that it is impossible to call a situation undignified; yet in our interpretation it would be an act of engagement with the situation to do so.
